# Hospital and surgeon volume *versus* outcomes after colorectal cancer surgery: umbrella review and meta-analysis

**DOI:** 10.1093/bjsopen/zrag074

**Published:** 2026-06-24

**Authors:** Justin Ho, Essam Rama, Alessandro Martinino, Francesco Giovinazzo

**Affiliations:** Department of Surgery, University of Cambridge, Addenbrooke’s Hospital, Cambridge, UK; Department of Surgery, Hinchingbrooke Hospital, Huntingdon, UK; Department of Surgery, Duke University, Durham, North Carolina, USA; Department of Surgery, Ospedale San Camillo, Treviso, Italy

**Keywords:** centralization, volume–outcome relationship, health services research

## Abstract

**Background:**

An inverse relationship between surgical volume and outcomes has been suggested, with higher-volume hospitals and surgeons achieving better results, prompting debate over the centralization of surgical services. However, minimum volume thresholds are unclear, and volume is a poor proxy for quality. Despite the significant global burden of colorectal cancer, the benefits of high-volume care remain uncertain. This umbrella review synthesized the evidence on volume–outcome associations in colorectal surgery.

**Methods:**

An umbrella review (PRISMA 2020) was conducted to evaluate systematic reviews and meta-analyses of the hospital/surgeon volume–outcome relationship in colorectal cancer. The Cochrane Library, PubMed, Embase, and MEDLINE were searched to 1 October 2025. Volume definitions and outcomes were extracted and meta-analysed by subgroup. A MeaSurement Tool to Assess Systematic Reviews 2 and Risk of Bias in Systematic Reviews were used for analysis of bias.

**Results:**

A total of 150 unique records was identified, with 10 systematic reviews meeting the inclusion criteria. High- *versus* low-volume hospitals demonstrated an inverse relationship in terms of postoperative mortality following resection for rectal cancer (fixed- and random-effects models: odds ratio 0.73, 95 per cent confidence interval 0.64 to 0.82), colon cancer (fixed-effect model: odds ratio 0.74, 0.70 to 0.78; random-effects model: odds ratio 0.75, 0.69 to 0.81), and colorectal cancer (fixed- and random-effects models: odds ratio 0.77, 0.67 to 0.88). High- *versus* low-volume surgeons demonstrated an inverse relationship with respect to postoperative mortality following resection for rectal cancer (fixed- and random-effects models: odds ratio 0.69, 0.59 to 0.81), colon cancer (fixed-effect model: odds ratio 0.70, 0.63 to 0.77; random-effects model: odds ratio 0.68, 0.55 to 0.85), and colorectal cancer (fixed- and random-effects models: odds ratio 0.67, 0.60 to 0.74). There were no consistent significant differences in rates of the secondary outcomes (anastomotic leak rate, permanent stoma formation, local recurrence rate, rate of abdominoperineal excision of the rectum).

**Conclusion:**

High-volume hospitals and surgeons are associated with both improved short- and long-term outcomes for patients undergoing colorectal cancer surgery. However, a specific cut-off definition for high- *versus* low-volume hospitals and surgeons is yet to be elucidated owing to the heterogeneity of existing volume definitions. Future studies are required to confirm a threshold for this dose–response relationship.

## Teaser

The volume–outcome relationship in colorectal cancer surgery remains controversial, with no agreed minimum volume thresholds. This umbrella review found that higher hospital and surgeon volumes were consistently associated with lower postoperative mortality across colorectal cancer subtypes. Higher surgeon volume showed the most consistent association with improved overall survival. No consistent benefit was observed for secondary outcomes including anastomotic leak rate, permanent stoma formation, local recurrence, or rate of abdominoperineal excision of the rectum.

## Introduction

An inverse volume–outcome relationship has been suggested for surgical procedures since 1979^1^. This assumes that higher-volume hospitals and surgeons tend to have lower (inverse) patient mortality rates and better outcomes than those with lower volumes. This has led to healthcare policymakers discussing whether there could be regionalization or centralization of surgical services to improve patient outcomes with respect to both morbidity and mortality. Since then, multiple studies have investigated the benefits of increased hospital and surgeon volumes for postoperative outcomes.

Despite investigations into purported minimum thresholds that achieve this volume–outcome connection, there is still significant debate around volume-based referral and whether patients should be referred selectively to high-volume institutions or surgeons. Those who support the idea that practice makes perfect and the intuitive logic that clinicians who perform the same procedure repetitively to achieve competence and specialization believe that individual procedures should be limited to centralized high-volume institutions^2^. Conversely, the suggestion is that low-volume institutions or surgeons would be associated with poorer patient outcomes. This is problematic as volume is a poor proxy for quality: high-volume providers with poor outcomes and low-volume providers with good outcomes can exist. Furthermore, critics of volume-based referral suggest that low-volume providers may experience a negative financial impact, with high-volume centres benefitting from managing patients from smaller-volume institutions^3^.

Colorectal cancer is the third most common diagnosis in both sexes in the USA, and is the second most common cause of cancer-related mortality worldwide^4^. Given this significant global burden, optimization of public health initiatives and organizations to improve patient outcomes is required. However, with colorectal cancer, the evidence for a volume–outcome relationship is blurred. Indeed, the threshold for what constitutes a high-volume hospital or surgeon has not been identified, and studies have reported mixed results regarding whether a true hospital or surgeon volume–outcome relationship exists.

The aim of this umbrella review was to systematically synthesize and evaluate evidence for a hospital and surgeon volume–outcome relationship for colorectal cancer procedures, without the assistance of artificial intelligence, as outlined in the TITAN 2025 guidelines^5^. This review sought to investigate the strength of the volume–outcome relationship, whether this association is clinically important, and whether there is any evidence of a dose–response threshold effect to support clinical and infrastructural decision-making in identifying a potential need for centralization.

## Methods

This umbrella review of systematic reviews, meta-analyses, and included literature was prepared and reported according to the PRISMA 2020 guidelines^6^ (*Table S1*), and registered prospectively with the Prospective Register of Systematic Reviews database on 9 September 2025 (PROSPERO 2025 identification number CRD420251143705). As this research involved literature that was already published, ethical approval was not required. This manuscript was prepared without the use of artificial intelligence.

### Search strategy

Databases were searched for peer-reviewed, systematic reviews and meta-analyses published from conception to 1 October 2025. These included the Cochrane Database of Systematic Reviews, PubMed, Embase, and MEDLINE. Reference lists of identified studies were also reviewed. The search strategy focused on colorectal cancer, hospital/surgeon volume, and the desired study design (*Fig. S1*).

### Eligibility criteria

The following inclusion criteria were used for all studies screened: adults undergoing colorectal cancer resection (reviews including mixed populations were eligible if colorectal outcomes were reported separately or clearly predominant); hospital and surgeon procedural volume (annual caseload) and, where applicable, cumulative surgeon experience; lower-*versus* higher-volume hospital and surgeon categories as defined in the included reviews/meta-analyses (no fixed cut-offs were imposed *a priori*); and systematic reviews and meta-analyses of observational or interventional primary studies that examined volume–outcome associations in colorectal cancer surgery across hospital and surgical departments. There were no language or country income restrictions.

Exclusion criteria were: adults undergoing other procedures with no relation to colorectal cancer; absence of indication of hospital and surgeon procedural volume (annual caseload), or cumulative surgeon experience; narrative reviews without systematic methods; single primary studies (unless embedded within eligible reviews); paediatric or non-oncological populations only; reviews without extractable effect estimates; and duplicate reviews with complete overlap (the most comprehensive/recent was retained).

### Study screening and selection

After obtaining the database results and removing duplicates, identified articles were imported into Rayyan^7^. Two reviewers (J.H. and E.R.) independently screened, with blinding, the titles and abstracts of identified articles that were relevant to the study inclusion criteria. Full texts were then imported and reviewed by the same two individuals for inclusion in this review. Any disagreements were initially resolved by further discussion, or adjudication by a third reviewer (A.M). when agreement could not be reached.

### Risk of bias

The methodological quality of the included systematic reviews was assessed independently by two reviewers (J.H. and E.R.) using two validated appraisal tools. Risk of Bias in Systematic Reviews (ROBIS)^8^ was applied to evaluate the risk of bias across the review process, whereas A MeaSurement Tool to Assess Systematic Reviews (AMSTAR) 2^9^ was used to assess overall methodological rigour, providing a comprehensive evaluation framework. Overlap assessment was conducted by generating a citation matrix of all original included primary manuscripts. The corrected covered area (CCA) was calculated from that matrix to quantify the degree of overlap.

### Data extraction, analysis, and synthesis

Data extraction was completed using a standardized data extraction template created *a priori*. Relevant manuscript characteristics and quantitative findings relating to the meta-analyses were extracted independently by two reviewers (J.H. and E.R.) and were cross-checked for accuracy/discrepancies by a third reviewer (A.M.). Any disagreements were resolved by discussion, or if necessary, by a fourth reviewer (F.G.). Where data were deemed inadequate, the primary research articles were consulted if possible.

Descriptive qualitative analyses of demographics for all included studies were undertaken using narrative synthesis. Definitions used for high and low volume in the included studies were extracted, and all outcomes compared in the two volume groups were also included if meta-analysed. Primary outcomes extracted included: 30-day mortality, inpatient/in-hospital mortality, intraoperative mortality, overall survival, and surgical morbidity. Secondary outcomes included: anastomotic leak rate, permanent stoma formation, local recurrence rate, and rate of abdominoperineal excision of the rectum (APER).

Data were split by tumour location into colon, rectal, and colorectal groups in accordance with the initial analyses. Hazard ratios (HRs) and odds ratios (ORs) with relevant numerical data derived from quantitative analyses were recorded. The purpose and main findings of each study were also identified.

### Meta-analysis

Data were pooled in a meta-analysis when at least two separate studies assessing whether there was a relationship between high or low volume and the outcome of interest were identified. Data for both hospital and surgeon volume analyses were analysed. Meta-analyses were carried out on eligible extracted data, and outcome relationships with volume were reported as ORs and HRs with 95% confidence intervals. Two-sided *P* < 0.050 was considered statistically significant. Meta-analyses were performed using the meta package from RStudio version 1.4.1717 (RStudio^®^, PBC, Boston, MA, USA). Heterogeneity was computed using *I^2^* statistics, and interpreted as follows: low (*I*^2^ < 25%), low to moderate (*I^2^* 25≤–<50%), moderate to substantial (*I*^2^ 50≤–<75%), or substantial (*I^2^* ≥ 75%)^10^. The inverse-variance method with both fixed- and random-effects models was used alongside the DerSimonian–Laird estimator for τ^2^ in random-effects analysis and the Jackson method for confidence interval of τ^2^ and τ. In these models, an overall OR or HR value of < 1 favours high volume and > 1 favours low volume. Inclusion of 1 within the 95% confidence interval suggests there is no significant association.

Given the strong correlation between surgeon and hospital volume, a hierarchical perspective was used and interaction-based interpretation framework in which surgeon volume was considered nested within institutional volume rather than an independent exposure. This approach acknowledged collinearity between surgeon and hospital volume, and avoided overattribution of effects to individual or institutional factors in isolation.

## Results

### Characteristics of included studies

**Fig. 1:**
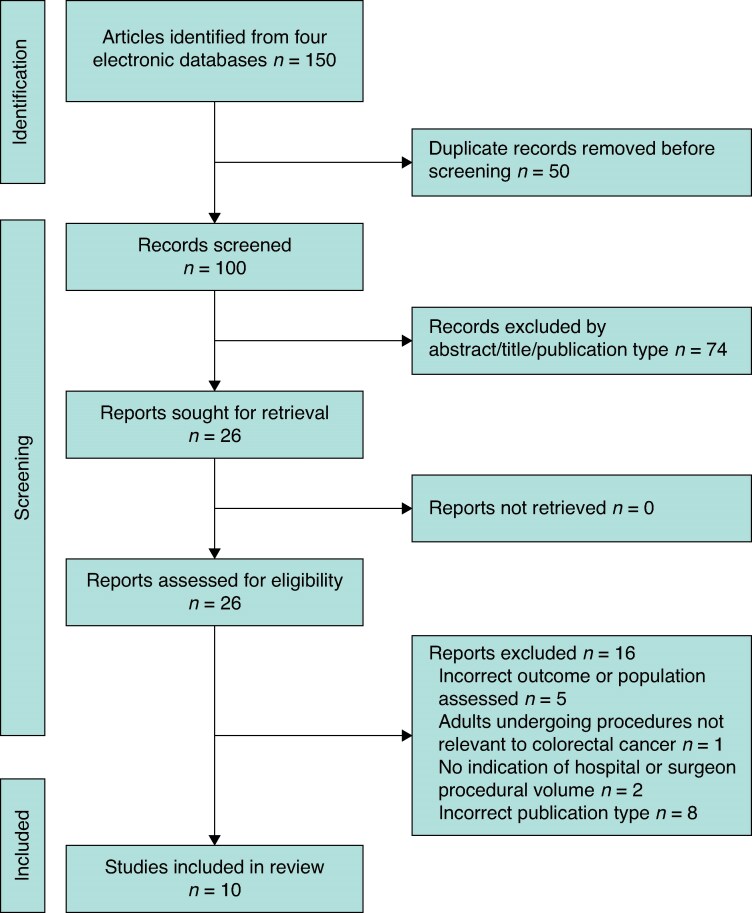
PRISMA diagram showing selection of articles for review

A total of 150 unique records was identified, of which 10 systematic reviews met the inclusion criteria for this umbrella review (*Fig. 1*). The characteristics and measured outcomes for each paper are reported in *Table S2.*

Across nine^11–18,19^ of the included papers, definitions of high and low hospital and surgeon volume were highly heterogeneous as they used the volume definitions of the original primary articles. A few papers^11,15,18^ demonstrated the exact overlap between definitions of high and low volume to illustrate this heterogeneity. For example, Iversen *et al*.^11^ included papers defining low-volume hospitals as performing 10–61 operations per year, and high-volume hospitals as 19–201 operations per year. van Gijn *et al*.^15^ used the original volume definitions in primary papers but reported a median cut-off point for high-volume hospitals of ≥ 126 procedures annually for colon cancer, ≥ 24 for rectal cancer, and ≥ 55 for the combined colorectal group. The median cut-off point for high-volume surgeons was ≥ 4 procedures annually for colon cancer and ≥ 17 for the combined colorectal group. Two reviews^13,18^ included studies that did not assign volume definitions *a priori* but divided the total caseload into segments of equal size (tertiles, quartiles). One paper^20^ introduced a novel definition for hospital volume based on the volume distribution of articles included. Articles in this particular review^20^ were classified as low hospital volumes if < 11 procedures per year, with all others classified into high-volume ≥ 11 rectal procedures per year.

The outcomes investigated with respect to high- *versus* low-volume groups included postoperative mortality (30 days, inpatient, in-hospital)^11,14–20^, intraoperative mortality^18^, overall survival (mortality, 5 years)^12–15,17,18,20^, surgical morbidity^18,20^, anastomotic leak rate^11,14,17,18^, permanent stoma formation^12,14,17^, local recurrence rate^14,17,18^, and rates of APER^14,17^. All ten studies^11–20^ included analyses of rectal cancer, nine^11–13,15–20^ included analyses of colon cancer, and eight^11–13,15–18,19^ included analyses of combined colorectal cancer. Hospital volume was investigated in nine studies^11–13,15–20^, and surgeon volume in seven studies^11,12,14–18^. Interestingly, analyses of both hospital and surgeon volume were conducted in only six studies^11,12,15–18^.

### Meta-analysis of hospital volume relationship with outcomes

A total of six studies^11,15–17,20,19^ of postoperative mortality after rectal cancer surgery, five studies^11,15–17,19^ of postoperative mortality after colon cancer surgery, four studies^11,15–17^ of postoperative mortality after colorectal cancer surgery, two studies^15,17^ of rectal and colon cancer overall survival, three studies^15,17,18^ of colorectal cancer overall survival, two studies^11,17^ of anastomotic leak after cancer surgery, and two studies^12,17^ of permanent stoma formation after rectal cancer surgery were selected on the basis that there were common outcomes measured in the primary papers available for meta-analysis.

**Fig. 2:**
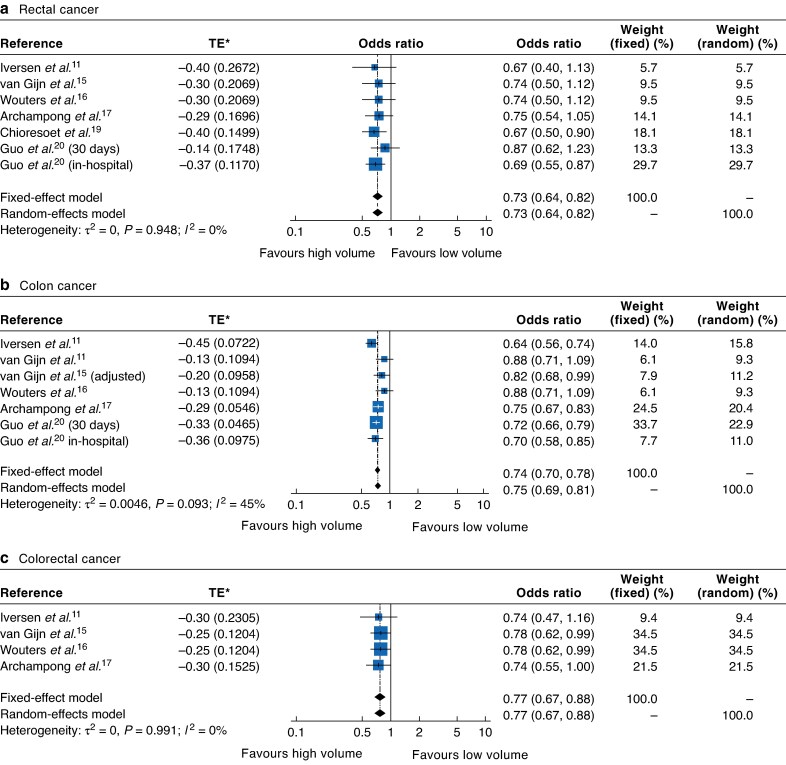
Forest plot of studies that evaluated the relationship between hospital volume and postoperative mortality following resection of rectal, colon, and colorectal cancer **a** Rectal, **b** colon, and **c** colorectal cancer. *Values in parentheses are standard errors. Odds ratios are shown with 95% confidence intervals. TE, treatment effect.

The relationships between high- *versus* low-volume hospitals with respect to postoperative mortality following resection of rectal, colon, and colorectal cancer were assessed. For rectal cancer, there was an inverse relationship in both fixed- and random-effects models (OR 0.73, 95% confidence interval (c.i.) 0.64 to 0.82) (*Fig. 2a*). For colon cancer, a similar effect was demonstrated in fixed-effect (OR 0.74, 0.70 to 0.78) and random-effects (OR 0.75, 0.69 to 0.81) models (*Fig. 2b*). This was further replicated for colorectal cancer in both models (OR 0.77, 0.67 to 0.88) (*Fig. 2c*).

**Fig. 3:**
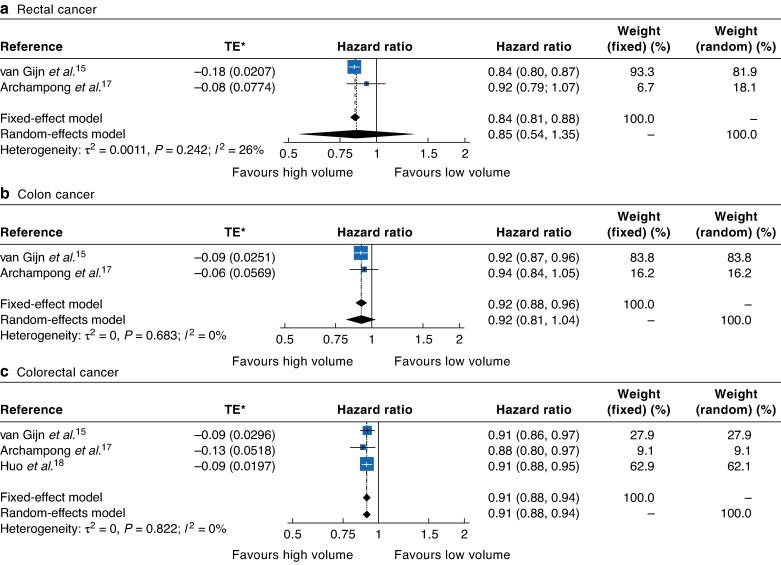
Forest plot of studies that evaluated the relationship between hospital volume and overall survival following resection of rectal, colon, and colorectal cancer **a** Rectal, **b** colon, and **c** colorectal cancer. *Values in parentheses are standard errors. Hazard ratios are shown with 95% confidence intervals. TE, treatment effect.

The relationships between high- *versus* low-volume hospitals in terms of overall survival following resection of rectal, colon, and colorectal cancer were assessed. For rectal cancer, there was an inverse relationship in a fixed-effect model (HR 0.84, 95% c.i. 0.81 to 0.88) but this was not significant in the random-effects analysis (HR 0.85, 0.54 to 1.35) (*Fig. 3a*). For colon cancer, an inverse relationship was demonstrated in a fixed-effect model (HR 0.92, 0.88 to 0.96) but this was not significant in a random-effects model (HR 0.92, 0.81 to 1.04) (*Fig. 3b*). Interestingly for colorectal cancer, there was a significant inverse relationship in both fixed- and random-effects models (HR 0.91, 0.88 to 0.94) (*Fig. 3c*).

The relationship between high- *versus* low-volume hospitals with respect to rates of anastomotic leak and permanent stoma formation following resection of rectal cancer were assessed. For anastomotic leak, there was no significant difference in outcome between high-and low-volume hospitals in fixed-effect (OR 1.21, 0.92 to 1.58) or random-effects (OR 1.21, 0.38 to 3.79) analyses. For permanent stoma formation, there was an inverse relationship in a fixed-effect model (OR 0.79, 0.75 to 0.82) but no significant difference between volume groups in random-effects analysis (OR 0.80, 0.37 to 1.76).

### Meta-analysis of surgeon volume relationship with outcomes

A total of three studies^11,14,17^ on postoperative mortality after rectal cancer surgery, four studies^11,15–17^ on postoperative mortality after colon cancer procedures, four studies^11,15–17^on postoperative mortality after colorectal cancer surgery, two studies each of rectal^14,17^ and colon^14,17^ cancer overall survival, three studies^15,17,18^ on colorectal cancer overall survival, two studies^14,18^ of rectal cancer recurrence rate, two studies^14,17^ of APER rate for rectal cancer, two studies^12,17^ of permanent stoma formation after rectal cancer surgery, and two studies^14,17^ of anastomotic leak after rectal cancer procedures were selected on the basis that there were common outcomes measured in the primary papers available for meta-analysis.

**Fig. 4:**
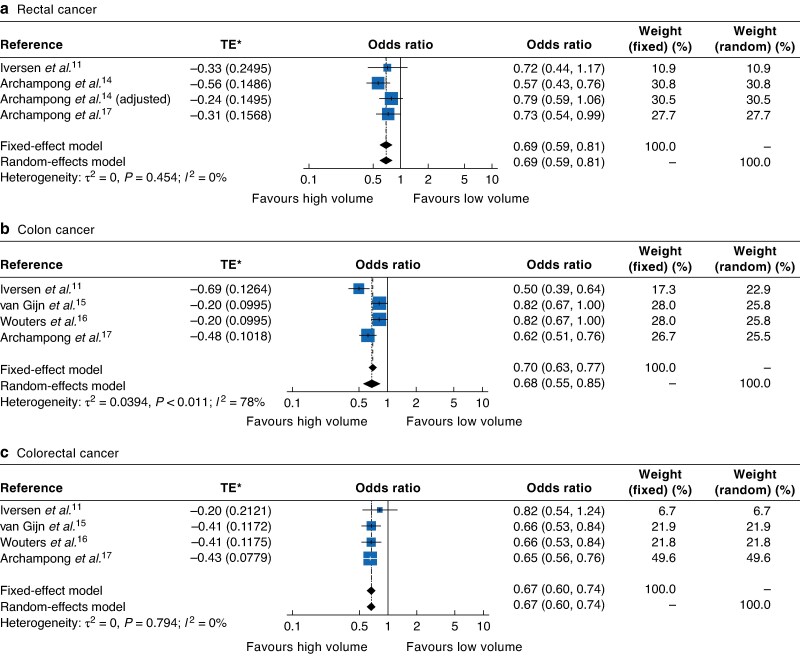
Forest plot of studies that evaluated the relationship between surgeon volume and postoperative mortality following resection of rectal, colon, and colorectal cancer **a** Rectal, **b** colon, and **c** colorectal cancer. *Values in parentheses are standard errors. Odds ratios are shown with 95% confidence intervals. TE, treatment effect.

The relationships between high- *versus* low-volume surgeons in terms of postoperative mortality following resection of rectal, colon, and colorectal cancer were assessed. For rectal cancer, there was an inverse relationship in both fixed- and random-effects models (OR 0.69, 95% c.i. 0.59 to 0.81) (*Fig. 4a*). For colon cancer, a similar effect was demonstrated in fixed-effect (OR 0.70, 0.63 to 0.77) and random-effects (OR 0.68, 0.55 to 0.85) models (*Fig. 4b*). This was further replicated for colorectal cancer in fixed- and random-effects models (OR 0.67, 0.60 to 0.74) (*Fig. 4c*).

**Fig. 5:**
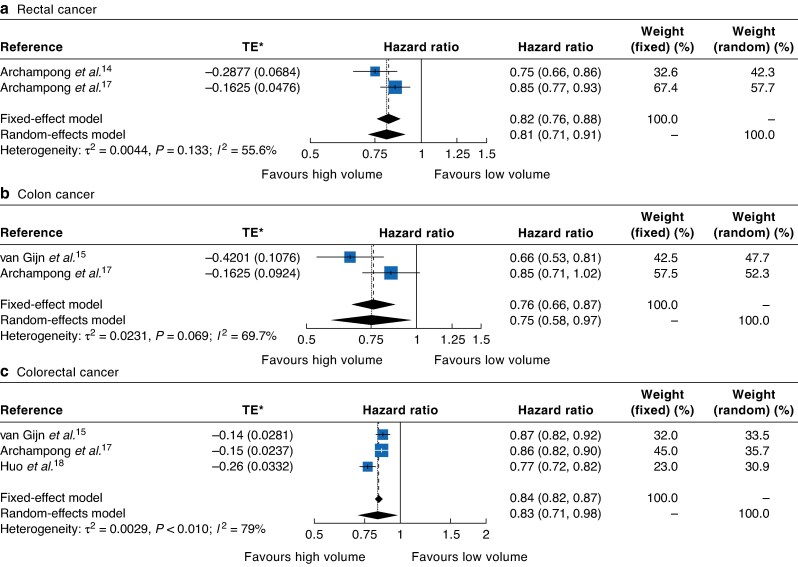
Forest plot of studies that evaluated the relationship between surgeon volume and overall survival following resection of rectal, colon, and colorectal cancer **a** Rectal, **b** colon, and **c** colorectal cancer. *Values in parentheses are standard errors. Hazard ratios are shown with 95% confidence intervals. TE, treatment effect.

The relationships between high- *versus* low-volume surgeons with respect to overall survival following rectal, colon, and colorectal cancer resection were assessed. For rectal cancer, an inverse relationship was demonstrated in both fixed-effect (HR 0.82, 95% c.i. 0.76 to 0.88) and random-effects (HR 0.81, 0.71to 0.91] models (*Fig. 5a***)**. For colon cancer, there was an inverse relationship in both fixed-effect (HR 0.76, 0.66 to 0.87) and random-effects (HR 0.75, 0.58 to 0.97) analyses (*Fig. 5b***)**. For colorectal surgery, there was an inverse relationship in both fixed-effect (HR 0.84, 0.82 to 0.87) and random-effects (HR 0.83, 0.71 to 0.98) models (*Fig. 5c*).

The relationship between high- *versus* low-volume surgeons in terms of rates of local recurrence following resection of rectal cancer were assessed. There was an inverse relationship in a fixed-effect model (OR 0.75, 0.65 to 0.87) but no significant difference between volume groups in random-effects analysis (OR 1.24, 0.00 to 2490.30). For APER rate following rectal cancer resection, there was an inverse relationship in a fixed-effect model (OR 0.62, 0.52 to 0.74) but no significant difference between volume groups in random-effects analysis (OR 0.62, 0.27 to 1.41). The relationships between high- *versus* low-volume surgeons in terms of rates of permanent stoma formation and anastomotic leak following resection of rectal cancer were assessed. For permanent stoma formation, there was a significant inverse relationship in both fixed- and random-effects models (OR 0.75, 0.66 to 0.85). For anastomotic leak, there was a significant inverse relationship in both fixed- and random-effects analyses (OR 0.68, 0.47 to 0.97) in high- compared with low-volume groups.

### Risk of bias and methodological quality

The combined ROBIS and AMSTAR 2 assessments highlighted inconsistencies in review quality. Most reviews had a low risk of bias in study eligibility criteria and study selection, but higher risk in data collection, appraisal, and particularly in synthesis and findings. Despite favourable ROBIS ratings overall, nearly all reviews were rated critically low by AMSTAR 2 owing to major methodological gaps, including lack of protocol registration, poor reporting of individual study exclusions, limited consideration of risk of bias, and inadequate reporting of funding and conflicts of interest. Overall, the results indicated adequacy of study selection processes but insufficient methodological rigour across most reviews (*Tables S3*, *S4* and *Figs S2*, *S3*).

### Citation matrix and CCA

Generation of a citation matrix showed that there were 110 unique primary index publications. There were 273 incidents of included publications in evidence synthesis across the included studies. The CCA was calculated as 16.5% which denotes very high overlap of primary studies across included reviews (*Table S5*).

## Discussion

The aim of this umbrella review was to systematically synthesize and evaluate evidence for a hospital and surgeon volume–outcome relationship in colorectal cancer surgery. The main findings were that high-volume hospitals and surgeons were consistently associated with lower postoperative mortality across all cancer types. There was improved overall survival following colorectal cancer resection for high-volume hospitals, and for all cancer resections for high-volume surgeons compared with low-volume counterparts. For anastomotic leak rate after rectal cancer resection, there was no significant difference between hospital volume groups. There were no consistent significant differences in rates of the other secondary outcomes (permanent stoma formation, local recurrence rate, APER rate). There was significant heterogeneity regarding volume definitions for high *versus* low volume. However, rather than representing independent predictors, surgeon and hospital volume are best interpreted as interacting components of surgical systems, with surgeon-level effects manifesting differently across institutional volume contexts.

This review suggests that both hospital and surgeon case volumes are associated with reduced mortality following resection of colon, rectal, and colorectal cancers. This is based on ten systematic reviews/meta-analyses spanning 20 years. The overall meta-analyses favour high-volume providers; however, not all analyses in the original included papers found a statistically significant association between volume and short- or long-term outcomes. This may be in part due to the lack of consensus regarding what constitutes a high- *versus* low-volume provider. For example, in the case of rectal cancer, National Institute for Health and Care Excellence guidelines^21^ suggest that hospitals carrying out major resection for rectal cancer should perform at least 10–20 operations a year, with individual surgeons expected to perform at least 5–10 operations annually. This threshold was found by the authors to have better outcomes than those of lower-volume providers in terms of overall survival, local recurrence, permanent stoma rates, and perioperative mortality, supporting centralization of services. Indeed, one^19^ of the included papers suggested that a similar threshold of 30 procedures per year may define a high-volume hospital for rectal resection. However, the issue with the current literature is the significant heterogeneity regarding volume definition. It is limited by different studies using different thresholds, and some did not treat volume as a continuous variable, leading to difficulty in generalizing consistent effects of case volume on outcomes. There is a lack of granularity regarding individual hospital and surgeon volume output; for example, a single surgeon operating at both high- and low-volume institutions. Re-pooling effect estimates from meta-analyses introduces potential meta-meta-analytic bias. This risk is heightened when included meta-analyses are heterogeneous, and vary in volume definitions and outcome specification. However, re-pooling was undertaken to summarize the direction and approximate magnitude of associations across manuscripts addressing a common underlying exposure–outcome relationship.

These meta-analyses demonstrated a significant inverse relationship between hospital and surgeon volume for all cancer types. The findings that higher surgeon volume improves both postoperative mortality and overall survival may suggest that this influences is stronger than that of the hospital volume. Hospital volume is linked with infrastructural efficiency in managing patients, postoperative management, and cross-specialty collaboration; surgeon volume is linked with appropriate patient selection, surgical technique, and operative decision-making^17^. One fundamental issue is that a high-volume surgeon will be by default operating in a high-volume hospital. Separating out the individual effects of both on outcomes is yet to be achieved. Interestingly, one study^22^ found that the interaction between surgeon and institutional volumes amplified the benefits. The included studies are unable to provide data on specialized low-volume surgeons working in high-volume hospitals or a high-volume surgeon performing multiple different procedures in an overall low-volume hospital. Another important consideration is delineation of the different types of cancer according to case complexity. For example, colon cancer can be divided into subcategories of T4/metastatic disease, emergency obstructed and perforated, and elective non-metastatic cancers. A fourth category of colon cancer in elderly frail patients may also be observed. Each of these demands different surgical skill paired with radiology, intensive care, oncology, and perioperative medical management. Indeed, high- *versus* low-volume institutions and surgeons may be differently equipped to deal with these categories, which contributes to the observed differences in outcomes.

The results also suggest that there is no difference in secondary outcomes between volume groups. The reason for this may be multifactorial: few studies were performed to examine these relationships, and longer-term outcomes may be less directly controlled by surgeon or hospital volume and more influenced by regional follow-up and long-term care after discharge. In the case of anastomotic leak, patient factors (body mass index, smoking status, nutritional status, tumour location), operative factors (sutured or stapled closure, surgeon skill on bowel apposition), and postoperative care (early recognition, packages of care, and follow-up) all contribute. Another potential explanation is that complex cases and high-risk patients in general may be referred to high-volume centres which can offset the volume advantage compared with low-volume providers, who may have a higher volume of patients with disease of lower complexity.

A limitation of this review is that the included papers focused predominantly on medical outcomes such as postoperative mortality. The notion of textbook outcomes is gaining popularity as a composite measure of combined outcome indicators, as it has been suggested to be of additional value over single outcome parameters in clinical auditing of surgical treatment^23^. However, when considering centralization of resources, it is important to consider other factors such as quality and disability-adjusted life-years, which are particularly important in volume-related analyses. This is because such factors play into a patient choice trade-off between centralization *versus* being treated close to home. Although the present data extraction was extensive, two studies^15,16^ had identical effect sizes for many of the outcomes assessed. Furthermore, the data from one paper^18^ were not included in the meta-analyses as effect sizes were reported as HRs and approximating to corresponding ORs was not considered appropriate. Most reviews were rated critically as being of low quality by AMSTAR, primarily because of the critical criteria (such as previous protocol registration) heavily weighting the rating even if all other aspects were acceptable. However, there were favourable ROBIS ratings overall, with 60% of papers having an overall low risk of bias. The CCA suggests that some duplication of primary studies was present and that there was a degree of overlap between patient data sets. However, the wide timespan of the included papers, which were exhaustive systematic reviews, supports the strength of the present conclusions. Future studies may consider whether changes in minimally invasive surgical techniques, enhanced recovery after surgery pathways, or advances in adjunctive treatment may modify applicability to contemporary practice.

## Supplementary Material

zrag074_Supplementary_Data

## Data Availability

The authors confirm that the data supporting the findings of this study are available within the article and its supplementary materials.
