# Supplementary material for: Hospital and surgeon volume versus outcomes after colorectal cancer surgery: umbrella review and meta-analysis
Source: BJS Open. 2026 Jun 24;10(3):zrag074. doi: 10.1093/bjsopen/zrag074 (PMC13293263; doi:10.1093/bjsopen/zrag074)
Supplement: zrag074_Supplementary_Data [file zrag074_supplementary_data.docx]

**Hospital and Surgeon Volume versus Outcomes after Colorectal Cancer Surgery:**

**Comprehensive Umbrella Review and Meta-Analysis**

Justin Ho MB BChir (Cantab)^1*^, Essam Rama MB BChir (Cantab)^2^, Alessandro Martinino MD, PhD^3^, Francesco Giovinazzo MD, PhD^4^

Affiliations

^1^Department of Surgery, University of Cambridge, Addenbrooke’s Hospital, Cambridge, UK

^2^Department of Surgery, Hinchingbrooke Hospital, Huntingdon, UK

^3^Department of Surgery, Duke University, Durham, North Carolina, USA

^4^Department of Surgery, Ospedale San Camillo, Treviso, Italy

*Corresponding author:

Justin Ho MB BChir (Cantab)

Department of Surgery

University of Cambridge, Addenbrooke’s Hospital, Hills Road, Cambridge, CB2 0QQ, UK

Email: [jkmh2@cam.ac.uk](mailto:jkmh2@cam.ac.uk)

**Supplementary Materials - Index**

| **Supplementary Figures and Tables** |  |
| --- | --- |
| Supplementary Table 1 | *pag. 2-6* |
| Supplementary Figure 1 | *pag. 7-8* |
| Supplementary Table 2 | *pag. 9-17* |
| Supplementary Table 3  Supplementary Figure 2  Supplementary Table 4  Supplementary Figure 3  Supplementary Table 5 | *pag. 18*  *pag. 19*  *pag. 20-21*  *pag. 22*  *pag. 23-24* |
|  |  |
|  |  |

**Supplementary Figures and Tables**

| **Section and Topic** | **Item #** | **Checklist item** | **Location where item is reported** |
| --- | --- | --- | --- |
| **TITLE** | | |  |
| Title | 1 | Identify the report as a systematic review. | 1 |
| **ABSTRACT** | | |  |
| Abstract | 2 | See the PRISMA 2020 for Abstracts checklist. | 2 |
| **INTRODUCTION** | | |  |
| Rationale | 3 | Describe the rationale for the review in the context of existing knowledge. | 3 |
| Objectives | 4 | Provide an explicit statement of the objective(s) or question(s) the review addresses. | 3 |
| **METHODS** | | |  |
| Eligibility criteria | 5 | Specify the inclusion and exclusion criteria for the review and how studies were grouped for the syntheses. | 4 |
| Information sources | 6 | Specify all databases, registers, websites, organisations, reference lists and other sources searched or consulted to identify studies. Specify the date when each source was last searched or consulted. | 4 |
| Search strategy | 7 | Present the full search strategies for all databases, registers and websites, including any filters and limits used. | Figure S1 |
| Selection process | 8 | Specify the methods used to decide whether a study met the inclusion criteria of the review, including how many reviewers screened each record and each report retrieved, whether they worked independently, and if applicable, details of automation tools used in the process. | 5 |
| Data collection process | 9 | Specify the methods used to collect data from reports, including how many reviewers collected data from each report, whether they worked independently, any processes for obtaining or confirming data from study investigators, and if applicable, details of automation tools used in the process. | 5 |
| Data items | 10a | List and define all outcomes for which data were sought. Specify whether all results that were compatible with each outcome domain in each study were sought (e.g. for all measures, time points, analyses), and if not, the methods used to decide which results to collect. | 5-6, Table 1 |
|  | 10b | List and define all other variables for which data were sought (e.g. participant and intervention characteristics, funding sources). Describe any assumptions made about any missing or unclear information. | 5-6, Table 1 |
| Study risk of bias assessment | 11 | Specify the methods used to assess risk of bias in the included studies, including details of the tool(s) used, how many reviewers assessed each study and whether they worked independently, and if applicable, details of automation tools used in the process. | 5 |
| Effect measures | 12 | Specify for each outcome the effect measure(s) (e.g. risk ratio, mean difference) used in the synthesis or presentation of results. | Table 1 |
| Synthesis methods | 13a | Describe the processes used to decide which studies were eligible for each synthesis (e.g. tabulating the study intervention characteristics and comparing against the planned groups for each synthesis (item #5)). | 5-6 |
|  | 13b | Describe any methods required to prepare the data for presentation or synthesis, such as handling of missing summary statistics, or data conversions. | 5-6 |
|  | 13c | Describe any methods used to tabulate or visually display results of individual studies and syntheses. | 5-6 |
|  | 13d | Describe any methods used to synthesize results and provide a rationale for the choice(s). If meta-analysis was performed, describe the model(s), method(s) to identify the presence and extent of statistical heterogeneity, and software package(s) used. | 5-6 |
|  | 13e | Describe any methods used to explore possible causes of heterogeneity among study results (e.g. subgroup analysis, meta-regression). | NA |
|  | 13f | Describe any sensitivity analyses conducted to assess robustness of the synthesized results. | NA |
| Reporting bias assessment | 14 | Describe any methods used to assess risk of bias due to missing results in a synthesis (arising from reporting biases). | NA |
| Certainty assessment | 15 | Describe any methods used to assess certainty (or confidence) in the body of evidence for an outcome. | NA |
| **RESULTS** | | |  |
| Study selection | 16a | Describe the results of the search and selection process, from the number of records identified in the search to the number of studies included in the review, ideally using a flow diagram. | Figure 1 |
|  | 16b | Cite studies that might appear to meet the inclusion criteria, but which were excluded, and explain why they were excluded. | Figure 1 |
| Study characteristics | 17 | Cite each included study and present its characteristics. | Table 1, 7 |
| Risk of bias in studies | 18 | Present assessments of risk of bias for each included study. | 9, Table S2/S3, Figure S2/S3 |
| Results of individual studies | 19 | For all outcomes, present, for each study: (a) summary statistics for each group (where appropriate) and (b) an effect estimate and its precision (e.g. confidence/credible interval), ideally using structured tables or plots. | 7-9, Figure 2abc, 3abc, 4abc, Figure 5abc |
| Results of syntheses | 20a | For each synthesis, briefly summarise the characteristics and risk of bias among contributing studies. | 7-9, Table S2/S3, Figure S2/S3 |
|  | 20b | Present results of all statistical syntheses conducted. If meta-analysis was done, present for each the summary estimate and its precision (e.g. confidence/credible interval) and measures of statistical heterogeneity. If comparing groups, describe the direction of the effect. | 7-9, Figure 2abc, 3abc, 4abc, Figure 5abc |
|  | 20c | Present results of all investigations of possible causes of heterogeneity among study results. | NA |
|  | 20d | Present results of all sensitivity analyses conducted to assess the robustness of the synthesized results. | NA |
| Reporting biases | 21 | Present assessments of risk of bias due to missing results (arising from reporting biases) for each synthesis assessed. | NA |
| Certainty of evidence | 22 | Present assessments of certainty (or confidence) in the body of evidence for each outcome assessed. | NA |
| **DISCUSSION** | | |  |
| Discussion | 23a | Provide a general interpretation of the results in the context of other evidence. | 9-11 |
|  | 23b | Discuss any limitations of the evidence included in the review. | 11 |
|  | 23c | Discuss any limitations of the review processes used. | 11 |
|  | 23d | Discuss implications of the results for practice, policy, and future research. | 11 |
| **OTHER INFORMATION** | | |  |
| Registration and protocol | 24a | Provide registration information for the review, including register name and registration number, or state that the review was not registered. | 4 |
|  | 24b | Indicate where the review protocol can be accessed, or state that a protocol was not prepared. | 4 |
|  | 24c | Describe and explain any amendments to information provided at registration or in the protocol. | NA |
| Support | 25 | Describe sources of financial or non-financial support for the review, and the role of the funders or sponsors in the review. | 11-12 |
| Competing interests | 26 | Declare any competing interests of review authors. | 11-12 |
| Availability of data, code and other materials | 27 | Report which of the following are publicly available and where they can be found: template data collection forms; data extracted from included studies; data used for all analyses; analytic code; any other materials used in the review. | 11-12 |

**Supplementary Table 1**: PRISMA 2020 table.

**MEDLINE and EMBASE**

1. Colorectal Neoplasms/ or Colorectal Surgery/ or Rectal Neoplasms/ or Colon Neoplasms/

2. (colorectal or colon or rectal or rectum).ti,ab.

3. (colectom* or proctectom* or proctom* or "anterior resection" or "abdominoperineal resection" or "total mesorectal excision" or TME).ti,ab.

4. 1 or 2 or 3

5. (hospital volume* or hospital caseload* or centre volume* or center volumxae* or institutional volume*).ti,ab.

6. (surgeon volume* or surgeon caseload* or surgeon case load* or surgeon experience or surgeon caseload*).ti,ab.

7. (procedure volume* or procedural volume* or case volume* or caseload* or volume outcome*).ti,ab.

8. (high-volume or high volume or low-volume or low volume or volume threshold*).ti,ab.

9. 5 or 6 or 7 or 8

10. (postoperativ* or postoperative or perioperative or intraoperative).ti,ab.

11. (complication* or surgical complication* or morbidity or mortality or "in-hospital mortality" or "30-day mortality" or "90-day mortality").ti,ab.

12. (surviv* or "overall survival" or "disease-free survival" or DFS or recurrence* or oncologic outcome* or cancer-specific survival or cancer specific survival).ti,ab.

13. (local recurrence or resection margin* or R0 or R1 or "circumferential resection margin" or CRM).ti,ab.

14. 10 or 11 or 12 or 13

15. Meta-Analysis/ or Systematic Review/

16. (meta-analy* or meta analy* or meta analy* or "systematic review" or "systematic reviews" or "pooled analysis" or "pooled estimate" or "review and meta-analysis").ti,ab.

17. 15 or 16

18. 4 and 9 and 14 and 17

**PUBMED**

("Colorectal Neoplasms"[Mesh] OR "Rectal Neoplasms"[Mesh] OR "Colon Neoplasms"[Mesh] OR colorectal[Title/Abstract] OR colon[Title/Abstract] OR rectal[Title/Abstract OR rectum[Title/Abstract] OR colectom*[Title/Abstract] OR proctectom*[Title/Abstract] OR proctom*[Title/Abstract] OR "anterior resection"[Title/Abstract] OR "abdominoperineal resection"[Title/Abstract] OR "total mesorectal excision"[Title/Abstract] OR TME[Title/Abstract])

AND

("Hospital Volume"[Title/Abstract] OR hospital volume*[Title/Abstract] OR hospital caseload*[Title/Abstract] OR centre volume*[Title/Abstract] OR center volume*[Title/Abstract] OR institutional volume*[Title/Abstract] OR surgeon volume*[Title/Abstract] OR surgeon caseload*[Title/Abstract] OR surgeon experience[Title/Abstract] OR procedure volume*[Title/Abstract] OR procedural volume*[Title/Abstract] OR case volume*[Title/Abstract] OR caseload*[Title/Abstract] OR volume-outcome*[Title/Abstract] OR high volume[Title/Abstract] OR high-volume[Title/Abstract] OR low volume[Title/Abstract] OR low-volume[Title/Abstract] OR volume threshold*[Title/Abstract])

AND

(postoperativ*[Title/Abstract] OR perioperative[Title/Abstract] OR intraoperative[Title/Abstract] OR complication*[Title/Abstract] OR morbidity[Title/Abstract] OR mortality[Title/Abstract] OR "in-hospital mortality"[Title/Abstract] OR "30-day mortality"[Title/Abstract] OR "90-day mortality"[Title/Abstract] OR survival[Title/Abstract] OR "overall survival"[Title/Abstract] OR "disease-free survival"[Title/Abstract] OR DFS[Title/Abstract] OR recurrence*[Title/Abstract] OR oncologic outcome*[Title/Abstract] OR "cancer-specific survival"[Title/Abstract] OR "local recurrence"[Title/Abstract] OR "circumferential resection margin"[Title/Abstract] OR CRM[Title/Abstract])

AND

("Systematic Review"[Publication Type] OR "Meta-Analysis"[Publication Type] OR meta-analy*[Title/Abstract] OR "systematic review"[Title/Abstract] OR "pooled analysis"[Title/Abstract] OR "review and meta-analysis"[Title/Abstract])

**Cochrane Database of Systematic Reviews**

(MeSH descriptor: [Colorectal Neoplasms] explode all trees OR MeSH descriptor: [Colon Neoplasms] explode all trees OR MeSH descriptor: [Rectal Neoplasms] explode all trees OR colorectal:ti,ab OR colon:ti,ab OR rectal:ti,ab OR colectom*:ti,ab OR proctectom*:ti,ab OR "anterior resection":ti,ab OR "abdominoperineal resection":ti,ab OR "total mesorectal excision":ti,ab)

AND

(hospital volume*:ti,ab OR hospital caseload*:ti,ab OR centre volume*:ti,ab OR surgeon volume*:ti,ab OR surgeon caseload*:ti,ab OR surgeon experience:ti,ab OR procedure volume*:ti,ab OR caseload*:ti,ab OR volume outcome*:ti,ab OR high volume:ti,ab OR low volume:ti,ab)

AND

(postoperative:ti,ab OR perioperative:ti,ab OR complication*:ti,ab OR morbidity:ti,ab OR mortality:ti,ab OR survival:ti,ab OR recurrence*:ti,ab OR "oncologic outcome*":ti,ab)

**Supplementary Figure 1:** Search criteria utilised in literature searches of selected databases.

| **Author (year)** | **Primary Studies Included (n)** | **Purpose** | **N of Patients (n), Patient Demographics** | **Definition of High vs Low Volume** | **Outcomes(s) from Meta Analysis** | **Cancer Condition** | **Metrics Odds Ratio or Hazard Ratio (95% CI)** | **p-value,**  **I^2^** | **Main Findings** |
| --- | --- | --- | --- | --- | --- | --- | --- | --- | --- |
| **Iversen A 2007**^11^ | 35 | To summarise current knowledge about the effect of hospital and surgeon characteristics on short-term outcomes following colorectal cancer surgery. | -, - | Among the studies, the cut-off point for low hospital caseload varied between 10 and 61 operations/year and for high hospital caseload between 19 and 201 operations/year. | Hospital caseload and postoperative mortality. | Colon | OR 0.64 (0.55 - 0.73) | <0.00001,  88.9% | The evidence suggests an association of high caseload and surgeon’s experience with improved post-operative morbidity and mortality for colorectal cancer. For colonic cancer, the analysis revealed that postoperative mortality is strongly influenced by hospital caseload and surgeon’s caseload. For rectal cancer, we did not observe any association between mortality and frequency of anastomotic leak. There exists a discrepancy between colon and rectal cancers results. |
|  |  |  |  |  | Hospital caseload and postoperative mortality. | Rectal | OR 0.67 (0.40 - 1.14) | 0.14,  71.9% |  |
|  |  |  |  |  | Hospital caseload and postoperative mortality. | Colorectal | OR 0.74 (0.47 - 1.16) | 0.19,  82.7% |  |
|  |  |  |  |  | Surgeon caseload and postoperative mortality. | Colon | OR 0.50 (0.39 - 0.64) | <0.00001,  85.4% |  |
|  |  |  |  |  | Surgeon caseload and postoperative mortality. | Rectal | OR 0.72 (0.44 - 1.17) | 0.18,  34.3% |  |
|  |  |  |  |  | Surgeon caseload and postoperative mortality. | Colorectal | OR 0.82 (0.54 - 1.24) | 0.34,  68% |  |
|  |  |  |  |  | Hospital caseload and frequency of anastomotic leak. | Rectal | OR 1.33 (0.89 - 1.99) | 0.16,  0% |  |
| **Iversen B 2007**^12^ | 34 | To summarise current knowledge about the effect of hospital and surgeon characteristics on long-term outcomes following colorectal cancer surgery. | -, - | Volume definitions as denoted by original articles | Hospital caseload and overall survival | Colon | OR 1.22 (1.16 - 1.28) | <0.00001,  0% | Long-term survival following colorectal cancer surgery improved significantly with increasing hospital caseload and surgeon volume. High hospital caseload also contributes to improved overall survival for colonic and rectal cancer, and to low frequency of permanent stoma in rectal cancer patient. |
|  |  |  |  |  | Hospital caseload and overall survival | Rectal | OR 1.38 (1.19 - 1.60) | <0.0001,  60.10% |  |
|  |  |  |  |  | Hospital caseload and overall survival | Colorectal | OR 1.17 (1.11 - 1.23) | <0.00001,  0% |  |
|  |  |  |  |  | Hospital caseload and frequency of permanent stoma formation | Rectal | OR 0.76 (0.68 - 0.85 | <0.00001,  41.2% |  |
|  |  |  |  |  | Surgeon caseload and overall survival | Colon | OR 1.25 (1.16 - 1.34) | <0.00001,  - |  |
|  |  |  |  |  | Surgeon caseload and overall survival | Rectal | OR 1.16 (0.88 - 1.54) | 0.3,  70% |  |
|  |  |  |  |  | Surgeon caseload and overall survival | Colorectal | OR 1.37 (0.94 - 1.99) | 0.1, 58.2% |  |
|  |  |  |  |  | Surgeon caseload and frequency of permanent stoma formation | Rectal | OR 0.75 (0.62 - 0.90) | 0.003,  10.6% |  |
| **Gruen 2009** ^13^ | 42 | To ascertain the strength and robustness of the association, if any, between hospital or clinician case volume and patient outcomes, whether this is clinically important and whether there is evidence of a threshold volume effect, above which better outcomes are observed? | -, - | Volume category cut points were determined post hoc through ranking providers (hospital or clinician) according to the number of procedures performed and then dividing into groups. | Hospital case volume and peri-operative mortality | Colon | OR 0.90 (0.88-0.92) | -, - | A significant volume effect was evident; with each doubling of hospital case volume, the odds of perioperative death decreased by 0.1 to 0.23. The authors calculated that between 10 and 50 patients per year, depending on cancer type, needed to be moved from a “low-volume” hospital to a “high-volume” hospital to prevent 1 additional volume-associated perioperative death |
|  |  |  |  |  | Hospital case volume and peri-operative mortality | Rectal | OR 1.07 (1.01 -1.14) | -, - |  |
|  |  |  |  |  | Hospital case volume and peri-operative mortality | Colorectal | OR 0.91 (0.89 - 0.93) | -, - |  |
| **Archampong 2010**^14^ | 10 | To clarify the relationship betwen surgical caseload and patient outcomes for patients undergoing rectal cancer surgery | 18,301, Mean 70 with mix of gender balance | Number of procedures performed in a year, or over a specified study period, and categorised as high volume or low volume according to the definition used in the original research. | Surgeon volume and unadjusted 30-day postoperative mortality | Rectal | OR 0.57 (0.43 - 0.77) | 0.0001, 0% | Patients of HV surgeons had significantly better overall survival: their risk of death was reduced by between 10% and 37%.We found some evidence that patients of HV surgeons had better 30-day mortality, reduced risk of local recurrence, stoma formation, and APER. |
|  |  |  |  |  | Surgeon volume and adjusted 30- day postoperative mortality (adjusted for case mix) | Rectal | OR 0.79 (0.59 - 1.06) | 0.11, 0% |  |
|  |  |  |  |  | Surgeon volume and overall survival | Rectal | HR 0.75 (0.65 - 0.86) | <0.0001, 18% |  |
|  |  |  |  |  | Surgeon volume and anastomotic leak rate | Rectal | OR 0.67 (0.38 - 1.17) | 0.16, 43% |  |
|  |  |  |  |  | Surgeon volume and local recurrence rate | Rectal | HR 2.4 (1.2 - 5.6) | 0.02, - |  |
|  |  |  |  |  | Surgeon volume and unadjusted permanent stoma formation | Rectal | OR 0.73 ( 0.58 - 0.91) | 0.006, 0% |  |
|  |  |  |  |  | Surgeon volume and adjusted permanent stoma formation | Rectal | OR 0.75 (0.64 - 0.88) | 0.0003, 0% |  |
|  |  |  |  |  | Surgeon volume and abdominoperineal excision (APER) rates | Rectal | OR 0.58 (0.45 - 0.76) | <0.0001, 67% |  |
|  |  |  |  |  | Surgeon volume and APER rates | **Non rectosigmoid** | OR 0.51 (0.28 - 0.91) | 0.03, 85% |  |
| **Van Gijn 2010** ^15^ | 23 | The objective of this review is to assess the volume-outcome relationship for colorectal cancer treatment. | 671,009 colon, 85,288 rectal, 80,481 colorectal cancer patients. 12,924 hospitals, 6,184 surgeons | Highest volume group compared to lowest volume group as defined in original studies. The median cut-off point of high volume hospitals is =>126 annual procedures for colon cancer, =>24 for rectal cancer and =>55 for colorectal cancer .  The median cut-off point of high volume surgeons is >=4 annual procedures for colon cancer and >=17 for colorectal cancer. | Hospital volume and postoperative mortality (unadjusted) | Colon | OR 0.88 (0.71 - 1.09) | 0.24, 87.1% | There is a clear and consistent relationship between high volume providers and an improved long term survival. This counts for both high volume hospitals and high volume surgeons. For the relation between volume and postoperative mortality, evidence is less convincing. |
|  |  |  |  |  | Hospital volume and postoperative mortality (adjusted) | Colon | OR 0.82 (0.68 - 0.99) | 0.039, - |  |
|  |  |  |  |  | Hospital volume and long term survival | Colon | HR 0.916 (0.872 - 0.962) | 0, 45.7% |  |
|  |  |  |  |  | Surgeon volume and postoperative mortality | Colon | OR 0.82 (0.675 - 0.997) | 0.046, - |  |
|  |  |  |  |  | Surgeon volume and long term survival | Colon | HR 0.657 (0.532 - 0.811) | <0.001, - |  |
|  |  |  |  |  | Hospital volume and postoperative mortality | Rectal | OR 0.744 (0.496 - 1.116) | 0.152, 76.7% |  |
|  |  |  |  |  | Hospital volume and long term survival | Rectal | HR 0.838 (0.805 - 0.873) | 0, 0% |  |
|  |  |  |  |  | Hospital volume and postoperative mortality | Colorectal | OR 0.781 (0.617 - 0.989) | 0.041, - |  |
|  |  |  |  |  | Hospital volume and long term survival | Colorectal | HR 0.912 (0.861 - 0.967) | 0.002, 37.7% |  |
|  |  |  |  |  | Surgeon volume and postoperative mortality | Colorectal | OR 0.665 (0.528 - 0.836) | 0, 45.6% |  |
|  |  |  |  |  | Surgeon volume and long term survival | Colorectal | HR 0.869 (0.823 - 0.919) | 0, 0% |  |
| **Wouters 2010**^16^ | 28 | To assess the relation between procedural volume and patient outcomes and whether cancer care in the Netherlands could be organised differently to assure high quality of care for all patients. | -, - | Volume definitions of original included studies. | Hospital volume and postoperative mortality | Rectal | OR 0.744 (0.496 - 1.116) | 0.153, - | On a population level, there is potential for improvement of outcome for cancer patients in the Netherlands by reducing var- iation in optimal treatment rates between hospitals. Colorectal cancer treatment should be provided in a specialized setting, with the right infrastructure, sufficient volume and adequate expertise. |
|  |  |  |  |  | Hospital volume and postoperative mortality | Colorectal | OR 0.781 (0.617 - 0.989) | 0.04, - |  |
|  |  |  |  |  | Hospital volume and postoperative mortality | Colon | OR 0.880 (0.710 - 1.090) | 0.242, - |  |
|  |  |  |  |  | Surgeon volume and postoperative mortality | Colon | OR 0.820 (0.675 - 0.997) | 0.046, - |  |
|  |  |  |  |  | Surgeon volume and postoperative mortality | Colorectal | OR 0.665 (0.528 - 0.837) | 0.001, - |  |
| **Archampong 2012**^17^ | 51 | Examine effects of hospital and surgeon volume on the outcomes of colorectal, colon and rectal cancer surgery. | 222,993 patients with colorectal cancer, 655,009 patients with colon cancer, and 65,726 patients with rectal cancer. | Volume definitions of original included studies. In studies with more than two stratified groups, the highest volume category was used for comparative analysis. | Hospital volume and overall survival | Colorectal | HR 0.88 (0.8-0.98) | 0.02, 93.97% | The results confirm the presence of a volume-outcome relationship in colorectal cancer surgery, based on hospital and surgeon caseload. The volume-outcome relationship appears somewhat stronger for the individual surgeon than for the hospital; particularly for overall survival and operative mortality. For death within five years of treatment, hospital volume appeared to be more beneficial in rectal cancer surgery than for colon cancer. |
|  |  |  |  |  |  | Colon | HR 0.94 (0.84 - 1.05) | 0.28, 96.9% |  |
|  |  |  |  |  |  | Rectal | HR 0.92 (0.79 - 1.07) | 0.26, 97.5% |  |
|  |  |  |  |  | Hospital volume and 30 day mortality | Colorectal | OR 0.74 (0.55-1.00) | 0.05, 95.2% |  |
|  |  |  |  |  |  | Colon | OR 0.75 (0.67 - 0.83) | <0.0001, 78% |  |
|  |  |  |  |  |  | Rectal | OR 0.75 (0.54 - 1.05) | 0.09, 75.1% |  |
|  |  |  |  |  | Hospital volume and five year local recurrence rate | Rectal | OR 0.70 (0.53 - 0.91) | 0.01, 54.8% |  |
|  |  |  |  |  | Hospital volume and anastomotic leak rate | Colon | OR 1.26 (0.66 - 2.41) | 0.48, 55.6% |  |
|  |  |  |  |  |  | Rectal | OR 1.11 (0.77 - 1.61) | 0.57, 57.8% |  |
|  |  |  |  |  | Hospital volume and permanent stoma rate | Rectal | OR 0.86 (0.75 -1.00) | 0.04, 69.9% |  |
|  |  |  |  |  | Hospital volume and abdominoperineal excision of rectum rate | Rectal | OR 0.80 (0.65 - 0.99) | 0.04, 75.7% |  |
|  |  |  |  |  | Surgeon volume and overall survival | Colorectal | HR 0.86 (0.82 - 0.90) | <0.0001, 39% |  |
|  |  |  |  |  |  | Colon | HR 0.85 (0.71 - 1.02) | 0.08, 82.8% |  |
|  |  |  |  |  |  | Rectal | HR 0.85 (0.78 - 0.94) | 0, 34.1% |  |
|  |  |  |  |  | Surgeon volume and 30 day mortality | Colorectal | OR 0.65 (0.56 - 0.76) | <0.0001, 9.27% |  |
|  |  |  |  |  |  | Colon | OR 0.62 (0.51 - 0.76) | <0.0001, 89.1% |  |
|  |  |  |  |  |  | Rectal | OR 0.73 (0.53 - 0.98) | 0.04, 27.8% |  |
|  |  |  |  |  | Surgeon volume and anastomotic leak rate | Colon | OR 0.58 (0.34 - 1.01) | 0.06, - |  |
|  |  |  |  |  |  | Rectal | OR 0.68 (0.43 - 1.08) | 0.11, 42.6% |  |
|  |  |  |  |  | Surgeon volume and permanent stoma rate | Rectal | OR 0.75 (0.62 - 0.89) | 0, 0% |  |
|  |  |  |  |  | Surgeon volume and abdominoperineal excision of the rectum | Rectal | OR 0.66 (0.52 - 0.84) | 0, 62.3% |  |
| **Huo 2017**^18^ | 47 | To clarify the association between colorectal surgery outcomes with hospital volume and surgeon volume. | 1,122,303 patients, 9,877 hospitals and 9,649 surgeons | For “low” hospital volume, cut-offs ranged from 5 or less operation per 5 years to 530 or less operations annually. For the “high” hospital volumes group, cut-off ranged from 6 or more annually to 2,623 or more operations annually.  For “low” surgeon volume, cut-offs ranged from 1 operation per 5 years, to 108 or less annually. For “high” surgeon volume, cut-offs ranged from 6–26 per 5 years, to 561 or more annually. | Hospital volume and 30 day mortality | Rectal | HR 0.81 (0.74 - 0.89) | <0.001, - | Higher hospital and surgeon volume resulted in reduced overall, in-hospital and intra-operative mortality. Post-operative complication rates depended on hospital not surgeon volume except with respect to anastomotic leak. High volume surgeons are associated with reduced recurrence rate and 5 year survival. The best outcomes occur in high volume hospitals with high volume surgeons. |
|  |  |  |  |  | Hospital volume and 30 day mortality | Colon | HR 0.67 (0.59 - 0.77) | <0.01, - |  |
|  |  |  |  |  | Hospital volume and 30 day mortality | Colorectal | HR 0.89 (0.82 - 0.96) | <0.001, - |  |
|  |  |  |  |  | Surgeon volume and 30 day mortality | Rectal | HR 0.89 (0.75–1.04) | -, - |  |
|  |  |  |  |  | Surgeon volume and 30 day mortality | Colon | HR 0.62 (0.53–0.72) | <0.001, - |  |
|  |  |  |  |  | Surgeon volume and 30 day mortality | Colorectal | HR 0.88 (0.82–0.94) | <0.001, - |  |
|  |  |  |  |  | Hospital volume and in hospital mortality | Rectal | HR 0.92 (0.65 - 0.99) | <0.05, - |  |
|  |  |  |  |  | Hospital procedure volume and in hospital mortality | Colon | HR 0.94 (0.89 - 0.98) | <0.001, - |  |
|  |  |  |  |  | Surgeon volume and in hospital mortality | Colon | HR 0.98 (0.97 - 0.99) | <0.01, - |  |
|  |  |  |  |  | Hospital volume and intraoperative mortality | Colon | HR 0.80 (0.76–0.85) | <0.001, - |  |
|  |  |  |  |  | Hospital volume and intraoperative mortality | Colorectal | HR 1.04 (0.86–1.27) | -, - |  |
|  |  |  |  |  | Surgeon volume and intraoperative mortality | Colorectal | HR 0.50 (0.40 - 0.62) | <0.001, - |  |
|  |  |  |  |  | Surgeon volume and 5 year survival | Colorectal | HR 0.77 (0.72 - 0.82) | <0.001, - |  |
|  |  |  |  |  | Hospital volume and 5 year survival | Rectal | HR 1.00 (0.99–1.01) | -, - |  |
|  |  |  |  |  | Hospital volume and 5 year survival | Colon | HR 1.09 (0.90–1.11) | -, - |  |
|  |  |  |  |  | Hospital procedure volume and 5 year survival | Colorectal | HR 0.91 (0.87–0.94) | <0.001, - |  |
|  |  |  |  |  | Surgeon volume and local recurrence | Rectal | HR 0.72 (0.62 - 0.83) | <0.001, - |  |
|  |  |  |  |  | Hospital volume and local recurrence | Rectal | HR 0.53 (0.42 - 0.62) | -, - |  |
|  |  |  |  |  | Hospital volume and post-op complications | Rectal | HR 0.94 (0.74 - 1.21) | -, - |  |
|  |  |  |  |  | Surgeon volume and post-op complications | Rectal | HR 0.80 (0.62 - 1.03) | -, - |  |
|  |  |  |  |  | Surgeon volume and anastomotic leak | Rectal | HR 0.59 (0.37 - 0.94) | <0.01, - |  |
|  |  |  |  |  | Hospital procedure volume and anastomotic leak | Rectal | HR 0.75 (0.58 - 0.97) | <0.05, - |  |
| **Chioreso 2018**^19^ | 21 | To determine if there is an association between hospital/surgeon volume and rectal cancer surgery outcomes among patients treated since 2000 | 155,810, Mean 59-67, more male than female. | Hospital volume was defined as the mean/number of rectal and/or rectosigmoid resections per year or over the study period in a specific hospital. Surgeon volume was defined as either the mean/number of resections performed by a surgeon per year or over the study period. **Articles were classified into low volume if ≤11 operations per year, while the rest were classified into high volume.** | Hospital volume and surgical morbidity (anastomotic leakage, abscess, iatrogenic, bleeding, peritonitis, stoma necrosis, stoma fistula, wound dehiscenece) | Rectal | OR 0.80 (0.70 - 0.93) | 0.003, 35% | The results of this study suggest that high hospital volume is associated with lower odds of surgical morbidity and post-operative mortality. Surgeon volume was not significantly associated with overall survival. |
|  |  |  |  |  | Hospital volume and post-operative mortality (30 day) | Rectal | OR 0.67 (0.50 - 0.90) | 0.008, 41% |  |
|  |  |  |  |  | Hospital volume and overall survival (follow up at 1, 3, or 5 years) | Rectal | OR 0.95 (0.91 - 1.00) | 0.07, 92% |  |
| **Guo 2025**^20^ | 45 | To explore the association between hospital volume and postoperative mortality in patients undergoing colon or rectum resection for colorectal cancer, with the aim of identifying a minimum threshold for high-volume hospitals. | 2,041,266 | Volume definitions of original included studies. | Hospital volume and 30 day postoperative mortality | Colon | OR 0.72 (0.65 - 0.78) | <0.1, 50.9% | Higher-volume hospitals reduce the risk of mortality after colorectal cancer surgery. Importantly, a threshold of 30 rectum resection per year may defined as a higher-volume hospital for rectal cancer. |
|  |  |  |  |  | Hospital volume and in hospital postoperative mortality | Colon | OR 0.70 (0.58 - 0.85) | <0.0001, 83% |  |
|  |  |  |  |  | Hospital volume and 30 day postoperative mortality | Rectal | OR 0.87 (0.62 - 1.23) | <0.01, 68% |  |
|  |  |  |  |  | Hospital volume and in hospital postoperative mortality | Rectal | OR 0.69 (0.55 - 0.87) | <0.0001, 90.5% |  |

**Supplementary Table 2:** Baseline characteristics of ten included studies

| Review | Phase 2 | | | | Phase 3 |
| --- | --- | --- | --- | --- | --- |
|  | Study eligibility criteria | Identification and selection of studies | Data collection and study appraisal | Synthesis and findings | Risk of bias in review |
| **Archampong 2010**^14^ | Low | Low | Low | Low | Low |
| **Archampong 2012**^17^ | Low | Low | Low | Low | Low |
| **Chioreso 2018**^19^ | Low | Low | Low | Low | Low |
| **Gruen 2009** ^13^ | Low | Low | Low | High | Low |
| **Guo 2025**^20^ | Low | Low | High | High | High |
| **Huo 2017**^18^ | Low | Low | Low | High | Low |
| **Iversen A 2007**^11^ | High | Low | High | High | High |
| **Iversen B 2007**^12^ | High | Low | High | High | High |
| **Van Gijn 2010** ^15^ | Low | Low | High | Low | Low |
| **Wouters 2010**^16^ | High | High | High | High | High |

**Supplementary Table 3**: ROBIS phase 2 and 3 risk of bias assessment for the included papers.


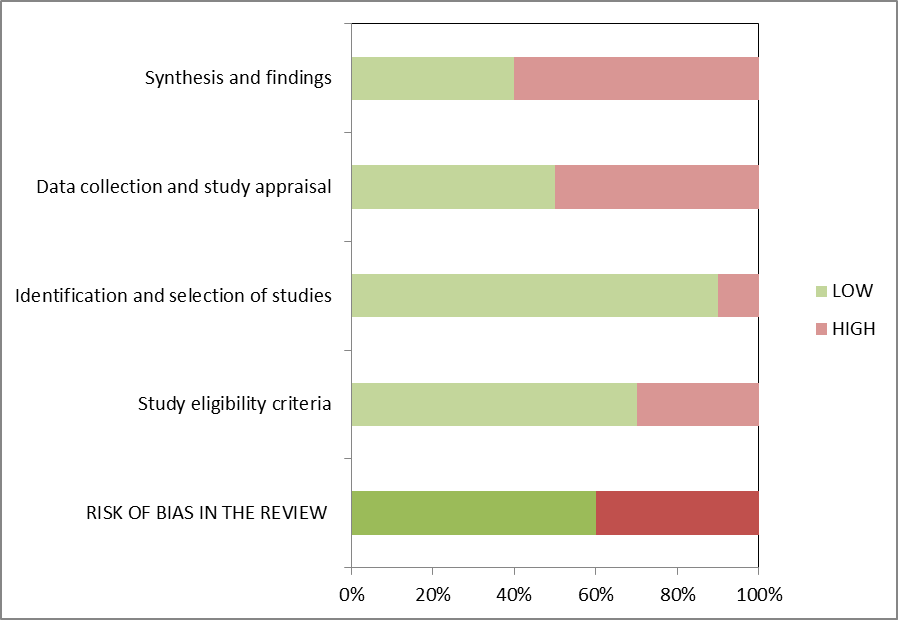


**Supplementary Figure 2:** Graphical representation of ROBIS domains categorised as low or high risk of bias for the included papers.

|  | **ITEMS** | | | | | | | | | | | |  |  |  |  | **Total**  **AMSTAR**  **Score** | **Final rating** |
| --- | --- | --- | --- | --- | --- | --- | --- | --- | --- | --- | --- | --- | --- | --- | --- | --- | --- | --- |
|  | **1** | **2** | **3** | **4** | **5** | **6** | **7** | **8** | **9** | **10** | **11** | **12** | **13** | **14** | **15** | **16** |  |  |
| **Archampong 2010**^14^ | Y | N | Y | Y | Y | Y | N | Y | Y | N | Y | N | Y | Y | Y | N | 11 | Critically Low |
| **Archampong 2012**^17^ | Y | N | Y | Y | Y | Y | Y | Y | Y | N | Y | Y | Y | Y | Y | N | 13 | Low |
| **Chioreso 2018**^19^ | Y | N | N | pY | Y | Y | N | Y | Y | N | Y | N | N | Y | Y | N | 9 | Critically Low |
| **Gruen 2009** ^13^ | Y | N | Y | Y | Y | Y | N | Y | Y | N | Y | Y | Y | Y | N | N | 11 | Critically Low |
| **Guo 2025**^20^ | Y | Y | N | Y | Y | Y | N | N | Y | N | Y | Y | Y | Y | Y | N | 11 | Low |
| **Huo 2017**^18^ | Y | N | N | pY | Y | Y | N | pY | N | N | N | N | N | N | N | Y | 6 | Critically Low |
| **Iversen A 2007**^11^ | Y | N | N | Y | N | N | N | N | N | N | Y | N | N | Y | Y | N | 5 | Critically Low |
| **Iversen B 2007**^12^ | Y | N | N | Y | N | N | N | N | N | N | Y | N | N | Y | Y | N | 5 | Critically Low |
| **Van Gijn 2010** ^15^ | Y | N | Y | Y | Y | Y | N | N | Y | N | Y | N | N | Y | Y | N | 9 | Critically Low |
| **Wouters 2010**^16^ | Y | N | N | N | N | N | N | N | N | N | Y | N | N | N | N | N | 2 | Critically Low |

**Supplementary Table 4**: AMSTAR 2 overall risk of bias assessment for the included papers.

1, PICO description; 2, protocol registered before the commencement of the review; 3, study design included in the review; 4, adequacy of the literature search; 5, two authors study selection; 6, two authors study extraction; 7, justification for excluding individual studies; 8, included studies descripted in detail; 9, risk of bias for the single studies being included in the review; 10, source of funding of primary studies; 11, appropriateness of meta-analytical methods; 12, impact of risk of bias of single studies on the results of the meta-analysis; 13, consideration of risk of bias when interpreting the results of the review; 14 explanation and discussion of the heterogeneity observed; 15, assessment of presence and likely impact of publication bias; 16, funding sources and conflict of interest declared.

**Abbreviations:** Y, yes; pY, partial yes; N, no.

**Footnotes:**

**High:** 0–1 non-critical weakness. The systematic review provides an accurate and comprehensive summary of the results of the available studies that address the question of interest.

**Moderate:** >1 non-critical weakness. The systematic review has more than one weakness, but no critical flaws. It may provide an accurate summary of the results of the available studies that were included in the review.

**Low:** 1 critical flaw with or without non-critical weaknesses. The review has a critical flaw and may not provide an accurate and comprehensive summary of the available studies that address the question of interest.

**Critically low:** >1 critical flaw with or without non-critical weaknesses. The review has more than one critical flaw and should not be relied on to provide an accurate and comprehensive summary of the available studies.


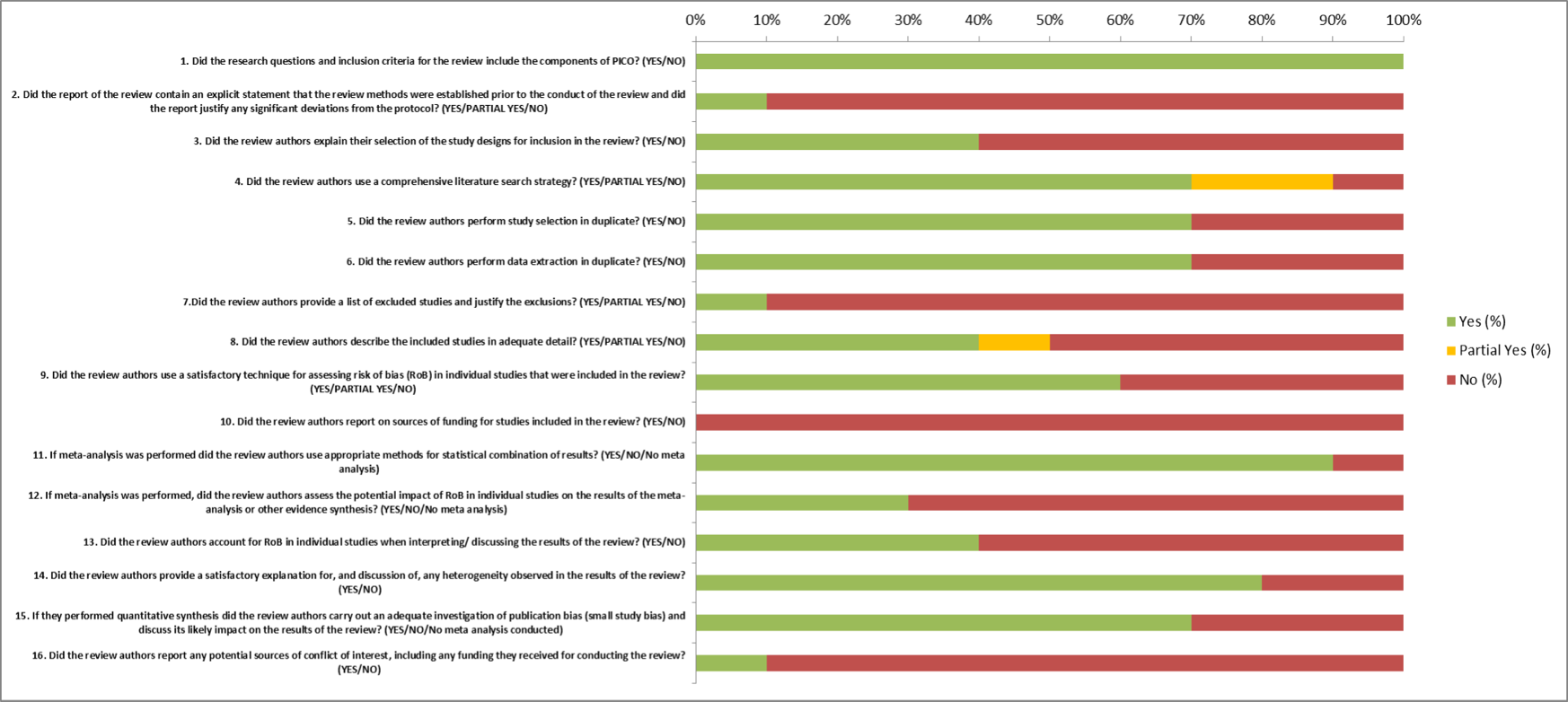


**Supplementary Figure 3:** Graphical representation of AMSTAR 2 risk of bias assessment for the included papers.

| **Author** | **Manuscript Title** | Archampong 2010 | Archampong 2012 | Chioreso 2018 | Gruen 2009 | Guo 2025 | Huo 2017 | Iversen 2006A | Iversen 2006B | Van Gijn 2010 | Wouters 2010 |
| --- | --- | --- | --- | --- | --- | --- | --- | --- | --- | --- | --- |
| **Aquina 2016** | High volume improves outcomes: the argument for centralization of rectal cancer surgery. |  |  |  |  |  |  |  |  |  |  |
| **Aubert 2020** | Impact of hospital volume on outcomes after emergency management of obstructive colon cancer: a nationwide study of 1957 patients |  |  |  |  |  |  |  |  |  |  |
| **Bader 2022** | Defining a minimum hospital volume threshold for minimally invasive colon cancer resections |  |  |  |  |  |  |  |  |  |  |
| **Baek 2013** | The association of hospital volume with rectal cancer surgery outcomes. International journal of colorectal disease. |  |  |  |  |  |  |  |  |  |  |
| **Begg 1998** | Impact of hospital volume on operative mortality for major cancer surgery |  |  |  |  |  |  |  |  |  |  |
| **Bilimoria 2008** | Directing surgical quality improvement initiatives: comparison of perioperative mortality and long-term survival for cancer surgery |  |  |  |  |  |  |  |  |  |  |
| **Billingsley 2007** | Surgeon and Hospital Characteristics as Predictors of Major Adverse Outcomes Following Colon Cancer Surgery: Understanding the Volume-Outcome Relationship |  |  |  |  |  |  |  |  |  |  |
| **Birkmeyer 2002** | Hospital Volume and Surgical Mortality in the United States |  |  |  |  |  |  |  |  |  |  |
| **Birkmeyer 2003** | Surgeon Volume and Operative Mortality in the United States |  |  |  |  |  |  |  |  |  |  |
| **Birkmeyer 2007** | Hospital volume and late survival after cancer surgery |  |  |  |  |  |  |  |  |  |  |
| **Borowski 2007** | Impact of surgeon volume and specialization on short-term outcomes in colorectal cancer surgery. |  |  |  |  |  |  |  |  |  |  |
| **Borowski 2010** | Volume-outcome analysis of colorectal cancer-related outcomes. |  |  |  |  |  |  |  |  |  |  |
| **Bos 2016** | No Difference in Overall Survival Between Hospital Volumes for Patients With Colorectal Cancer in The Netherlands. |  |  |  |  |  |  |  |  |  |  |
| **Boudorakis 2009** | Evolution of the surgeon-volume, patient-outcome relationship |  |  |  |  |  |  |  |  |  |  |
| **Callahan 2003** | Influence of Surgical Subspecialty Training on In-Hospital Mortality for Gastrectomy and Colectomy Patients |  |  |  |  |  |  |  |  |  |  |
| **Carter 1995** | Consultant surgeons and pathologists of the lothian and borders health boards. Lothian and borders large bowel cancer project: immediate outcome after surgery. |  |  |  |  |  |  |  |  |  |  |
| **COLOR Study Group** | Impact of hospital case volume on short-term outcome after laparoscopic operation for colonic cancer |  |  |  |  |  |  |  |  |  |  |
| **Comber 2012** | Quality of rectal cancer surgery and its relationship to surgeon and hospital caseload: a population-based study. |  |  |  |  |  |  |  |  |  |  |
| **Debes 2008** | Curative rectal cancer surgery in a low-volume hospital: A quality assessment |  |  |  |  |  |  |  |  |  |  |
| **Dimick 2003** | Hospital volume and surgical outcomes for elderly patients with colorectal cancer in the United States |  |  |  |  |  |  |  |  |  |  |
| **Dorrance 2000** | Effect of surgeon specialty interest on patient outcome after potentially curative colorectal cancer surgery |  |  |  |  |  |  |  |  |  |  |
| **Drolet 2011** | Elective Resection of Colon Cancer by High-Volume Surgeons Is Associated with Decreased Morbidity and Mortality |  |  |  |  |  |  |  |  |  |  |
| **Elferink 2010A** | Disparities in quality of care for colon cancer between hospitals in the Netherlands |  |  |  |  |  |  |  |  |  |  |
| **Elferink 2010B** | Variation in treatment and outcome of patients with rectal cancer by region, hospital type and volume in the Netherlands |  |  |  |  |  |  |  |  |  |  |
| **Engel 2005A** | Operative mortality after colorectal resection in the Netherlands |  |  |  |  |  |  |  |  |  |  |
| **Engel 2005B** | Influence of hospital volume on local recurrence and survival in a population sample of rectal cancer patients |  |  |  |  |  |  |  |  |  |  |
| **Finlayson 2003** | Hospital Volume and Operative Mortality in Cancer Surgery: A National Study |  |  |  |  |  |  |  |  |  |  |
| **Galandiuk 2006** | Differences and similarities between rural and urban operations |  |  |  |  |  |  |  |  |  |  |
| **Gordon 1999** | Complex gastrointestinal surgery: impact of provider experience on clinical and economic outcomes |  |  |  |  |  |  |  |  |  |  |
| **Gort 2010** | Actionable indicators for short and long term outcomes in rectal cancer |  |  |  |  |  |  |  |  |  |  |
| **Guller 2017** | Lower hospital volume is associated with higher mortality after oesophageal, gastric, pancreatic and rectal cancer resection |  |  |  |  |  |  |  |  |  |  |
| **Hannan 1989** | Investigation of the relationship between volume and mortality for surgical procedures performed in New York State hospitals. |  |  |  |  |  |  |  |  |  |  |
| **Hannan 2002** | The influence of hospital and surgeon volume on in-hospital mortality for colectomy, gastrectomy, and lung lobectomy in patients with cancer |  |  |  |  |  |  |  |  |  |  |
| **Harling 2005** | Hospital volume and outcome of rectal cancer surgery in Denmark 1994–99 |  |  |  |  |  |  |  |  |  |  |
| **Harmon 1999** | Hospital volume can serve as a surrogate for surgeon volume for achieving excellent outcomes in colorectal resection. |  |  |  |  |  |  |  |  |  |  |
| **Hermanek 1999** | Impact of surgeon's technique on outcome after treatment of rectal carcinoma |  |  |  |  |  |  |  |  |  |  |
| **Ho 2006** | Trends in hospital and surgeon volume and operative mortality for cancer surgery |  |  |  |  |  |  |  |  |  |  |
| **Hodgson 2003** | Relation of Hospital Volume to Colostomy Rates and Survival for Patients With Rectal Cancer |  |  |  |  |  |  |  |  |  |  |
| **Hohenberger 2013** | Volume and outcome in rectal cancer surgery: the importance of quality management. |  |  |  |  |  |  |  |  |  |  |
| **Holm 1997** | Influence of hospital- and surgeon-related factors on outcome after treatment of rectal cancer with or without preoperative radiotherapy. |  |  |  |  |  |  |  |  |  |  |
| **Jonker 2017A** | The influence of hospital volume on long-term oncological outcome after rectal cancer surgery |  |  |  |  |  |  |  |  |  |  |
| **Jonker 2017B** | The impact of hospital volume on perioperative outcomes of rectal cancer |  |  |  |  |  |  |  |  |  |  |
| **Kee 1999** | Influence of hospital and clinician workload on survival from colorectal cancer: cohort study |  |  |  |  |  |  |  |  |  |  |
| **Khuri 1999** | Relation of Surgical Volume to Outcome in Eight Common Operations: results from the VA NAtional Surgical Quality Improvement Program |  |  |  |  |  |  |  |  |  |  |
| **Ko 2002** | Are high-volume surgeons and hospitals the most important predictors of in-hospital outcome for colon cancer resection? |  |  |  |  |  |  |  |  |  |  |
| **Kolfschoten 2011** | Variation in case-mix between hospitals treating colorectal cancer patients in the Netherlands. |  |  |  |  |  |  |  |  |  |  |
| **Kressner 2009** | The Impact of Hospital Volume on Surgical Outcome in Patients with Rectal Cancer. |  |  |  |  |  |  |  |  |  |  |
| **Kuhry 2005** | Impact of hospital case volume on short-term outcome after laparoscopic operation for colonic cancer. |  |  |  |  |  |  |  |  |  |  |
| **Kuwabara 2009** | Impact of Hospital Case Volume on the Quality of Laparoscopic Colectomy in Japan |  |  |  |  |  |  |  |  |  |  |
| **Kwan 2008** | Population-Based Information on Emergency Colorectal Surgery and Evaluation on Effect of Operative Volume on Mortality |  |  |  |  |  |  |  |  |  |  |
| **LCCSC 2023** | Postoperative outcomes of right hemicolectomy for cancer in 11 countries of Latin America: A multicentre retrospective study |  |  |  |  |  |  |  |  |  |  |
| **Leonard 2014** | Effect of hospital volume on quality of care and outcome after rectal cancer surgery. |  |  |  |  |  |  |  |  |  |  |
| **Lin 2006** | Hospital volume and inpatient mortality after cancer-related gastrointestinal resections: the experience of an Asian country |  |  |  |  |  |  |  |  |  |  |
| **Liu 2015** | Association of surgeon volume and hospital volume with the outcome of patients receiving definitive surgery for colorectal cancer: A nationwide population-based study. |  |  |  |  |  |  |  |  |  |  |
| **Luft 1987** | The volume-outcome relationship: practice-makes-perfect or selective-referral patterns? |  |  |  |  |  |  |  |  |  |  |
| **Manchon Walsh 2011** | Variability in the quality of rectal cancer care in public hospitals in Catalonia (Spain): Clinical audit as a basis for action |  |  |  |  |  |  |  |  |  |  |
| **Martling 2002** | The surgeon as a prognostic factor after the introduction of total mesorectal excision in the treatment of rectal cancer. |  |  |  |  |  |  |  |  |  |  |
| **Marusch 2001A** | Hospital caseload and the results achieved in patients with rectal cancer |  |  |  |  |  |  |  |  |  |  |
| **Marusch 2001B** | Effect of caseload on the short-term outcome of colon surgery: results of a multicenter study |  |  |  |  |  |  |  |  |  |  |
| **Marwan 2010** | The Rate of Abdominoperineal Resections for Rectal Cancer in the State of Victoria, Australia: A Population-Based Study |  |  |  |  |  |  |  |  |  |  |
| **Matthiessen 2006** | Population-based study of risk factors for postoperative death after anterior resection of the rectum |  |  |  |  |  |  |  |  |  |  |
| **McArdle 2004** | Influence of volume and specialization on survival following surgery for colorectal cancer |  |  |  |  |  |  |  |  |  |  |
| **McGrath 2005** | Surgeon and hospital volume and the management of colorectal cancer patients in Australia |  |  |  |  |  |  |  |  |  |  |
| **Mella 1997** | Population-based audit of colorectal cancer management in two UK health regions |  |  |  |  |  |  |  |  |  |  |
| **Meyerhardt 2003** | Association of Hospital Procedure Volume and Outcomes in Patients with Colon Cancer at High Risk for Recurrence |  |  |  |  |  |  |  |  |  |  |
| **Meyerhardt 2004** | Impact of Hospital Procedure Volume on Surgical Operation and Long-Term Outcomes in High-Risk Curatively Resected Rectal Cancer: Findings From the Intergroup 0114 Study |  |  |  |  |  |  |  |  |  |  |
| **Morris 2007** | Surgical volume influences survival in patients undergoing resections for stage II colon cancers |  |  |  |  |  |  |  |  |  |  |
| **Mroczkowski 2011** | Low-volume centre vs high-volume: the role of a quality assurance programme in colon cancer surgery |  |  |  |  |  |  |  |  |  |  |
| **Nathan 2015** | Hospital volume, complications, and cost of cancer surgery in the elderly |  |  |  |  |  |  |  |  |  |  |
| **Ng 2006** | Subspecialisation and its Effect on the Management of Rectal Cancer |  |  |  |  |  |  |  |  |  |  |
| **Oliphant 2014** | The impact of surgical specialisation on survival following elective colon cancer surgery |  |  |  |  |  |  |  |  |  |  |
| **Ortiz 2016** | Effect of hospital caseload on long-term outcome after standardization of rectal cancer surgery in the Spanish Rectal Cancer Project. |  |  |  |  |  |  |  |  |  |  |
| **Parry 1999** | Influence of volume of work on the outcome of treatment for patients with colorectal cancer. |  |  |  |  |  |  |  |  |  |  |
| **Platell 2003** | Dose surgical sub-specialization influence survival in patients with colorectal cancer? |  |  |  |  |  |  |  |  |  |  |
| **Porter 1998** | Surgeon-related factors and outcome in rectal cancer |  |  |  |  |  |  |  |  |  |  |
| **Prystowsky 2002** | Patient outcomes for segmental colon resection according to surgeon's training, certification, and experience |  |  |  |  |  |  |  |  |  |  |
| **Ptok 2007** | Influence of hospital volume on the frequency of abdominoperineal resections and long-term oncological outcomes in low rectal cancer |  |  |  |  |  |  |  |  |  |  |
| **Purves 2005** | Relationship between surgeon caseload and sphincter preservation in patients with rectal cancer |  |  |  |  |  |  |  |  |  |  |
| **Rabeneck 2004** | Surgical Volume and Long-Term Survival Following Surgery for Colorectal Cancer in the Veterans Affairs Health-Care System |  |  |  |  |  |  |  |  |  |  |
| **Reames 2014** | Hospital Volume and Operative Mortality in the Modern Era |  |  |  |  |  |  |  |  |  |  |
| **Renzulli 2006** | The influence of the surgeon's and the hospital's caseload on survival and local recurrence after colorectal cancer surgery |  |  |  |  |  |  |  |  |  |  |
| **Richardson 2013** | Surgeon knowledge contributes to the relationship between surgeon volume and patient outcomes in rectal cancer. |  |  |  |  |  |  |  |  |  |  |
| **Riley 1985** | Outcomes of surgery among the Medicare aged: surgical volume and mortality. |  |  |  |  |  |  |  |  |  |  |
| **Rogers 2006** | Relation of surgeon and hospital volume to processes and outcomes of colorectal cancer surgery. |  |  |  |  |  |  |  |  |  |  |
| **Schrag 2000** | Influence of Hospital Procedure Volume on Outcomes Following Surgery for Colon Cancer |  |  |  |  |  |  |  |  |  |  |
| **Schrag 2002** | Hospital and Surgeon Procedure Volume as Predictors of Outcome Following Rectal Cancer Resection |  |  |  |  |  |  |  |  |  |  |
| **Schrag 2003** | Surgeon volume compared to hospital volume as a predictor of outcome following primary colon cancer resection |  |  |  |  |  |  |  |  |  |  |
| **Sheetz 2019** | Association of Discretionary Hospital Volume Standards for High-risk Cancer Surgery With Patient Outcomes and Access, 2005-2016 |  |  |  |  |  |  |  |  |  |  |
| **Simons 1997** | Variations in treatment of rectal cancer: The influence of hospital type and caseload |  |  |  |  |  |  |  |  |  |  |
| **Simunovic 2000** | Hospital procedure volume and teaching status do not influence treatment and outcome measures of rectal cancer surgery in a large general population |  |  |  |  |  |  |  |  |  |  |
| **Simunovic 2006** | Influence of hospital characteristics on operative death and survival of patients after major cancer surgery in Ontario |  |  |  |  |  |  |  |  |  |  |
| **Sjovall 2007** | Colon cancer management and outcome in relation to individual hospitals in a defined population |  |  |  |  |  |  |  |  |  |  |
| **Smith 2003** | Evidence of the effect of ‘specialization’ on the management, surgical outcome and survival from colorectal cancer in Wessex |  |  |  |  |  |  |  |  |  |  |
| **Stocchi 2001** | Impact of Surgical and Pathologic Variables in Rectal Cancer: A United States Community and Cooperative Group Report |  |  |  |  |  |  |  |  |  |  |
| **Tebe 2017** | Towards the centralization of digestive oncologic surgery: changes in activity, techniques and outcome |  |  |  |  |  |  |  |  |  |  |
| **Truong 2008** | The impact of hospital volume on the number of nodes retrieved and outcome in colorectal cancer. |  |  |  |  |  |  |  |  |  |  |
| **Turner 2020** | Comparison of Survival of Stage I-III Colon Cancer by Travel Distance and Hospital Volume |  |  |  |  |  |  |  |  |  |  |
| **Tustumi 2022** | The impact of the institutional abdominoperineal resections volume on short-term outcomes and expenses: a nationwide study |  |  |  |  |  |  |  |  |  |  |
| **Urbach 2003** | Differences in operative mortality between high- and low-volume hospitals in Ontario for 5 major surgical procedures: estimating the number of lives potentially saved through regionalization |  |  |  |  |  |  |  |  |  |  |
| **Urbach 2004** | Does it matter what a hospital is “high volume” for? Specificity of hospital volume-outcome associations for surgical procedures: analysis of administrative data |  |  |  |  |  |  |  |  |  |  |
| **van Erning 2013** | No difference between lowest and highest volume hospitals in outcome after colorectal cancer surgery in the southern Netherlands. |  |  |  |  |  |  |  |  |  |  |
| **Walther 2022** | Relationships between multiple patient safety outcomes and healthcare and hospital-related risk factors in colorectal resection cases: cross-sectional evidence from a nationwide sample of 232 German hospitals |  |  |  |  |  |  |  |  |  |  |
| **Wasif 2018** | Does Improved Mortality at Low- and Medium-Volume Hospitals Lead to Attenuation of the Volume to Outcomes Relationship for Major Visceral Surgery? |  |  |  |  |  |  |  |  |  |  |
| **Wibe 2005** | Effect of hospital caseload on long-term outcome after standardization of rectal cancer surgery at a national level |  |  |  |  |  |  |  |  |  |  |
| **Wirth 2022** | Does hospital volume affect outcomes after abdominal cancer surgery: an analysis of Swiss health insurance claims data |  |  |  |  |  |  |  |  |  |  |
| **Yasunaga 2009** | Volume-outcome relationship in rectal cancer surgery: a new perspective. |  |  |  |  |  |  |  |  |  |  |
| **Yeo 2017** | Surgeon Annual and Cumulative Volumes Predict Early Postoperative Outcomes after Rectal Cancer Resection |  |  |  |  |  |  |  |  |  |  |
| **Yun 2012** | The influence of hospital volume and surgical treatment delay on long-term survival after cancer surgery. |  |  |  |  |  |  |  |  |  |  |
| **Zhang 2007** | Patient characteristics and hospital quality for colorectal cancer surgery |  |  |  |  |  |  |  |  |  |  |
| **Zheng 2014** | Hospital center effect for laparoscopic colectomy among elderly stage I-III colon cancer patients |  |  |  |  |  |  |  |  |  |  |

**Supplementary Table 5**: Correlation matrix to evaluate overlap between included index publications across the ten included papers.

CCA (Corrected Covered Area)=(𝑁−𝑟)/(𝑟×𝑐)−𝑟, where *N* is the total number of included publications in evidence synthesis (this is the sum of the green highlighted boxes in the citation matrix); *r* is the number of rows (number of index publications); and *c* is the number of columns (number of included reviews).
